# Deaths in a Modern Cohort of Extremely Preterm Infants From the Preterm Erythropoietin Neuroprotection Trial

**DOI:** 10.1001/jamanetworkopen.2021.46404

**Published:** 2022-02-07

**Authors:** Sandra E. Juul, Thomas R. Wood, Bryan A. Comstock, Krystle Perez, Semsa Gogcu, Mihai Puia-Dumitrescu, Sara Berkelhamer, Patrick J. Heagerty

**Affiliations:** 1 Division of Neonatology, Department of Pediatrics, University of Washington, Seattle; 2 Department of Biostatistics, University of Washington, Seattle; 3 Division of Neonatology, Pediatrics, Wake Forest School of Medicine, Winston-Salem, North Carolina

## Abstract

**Importance:**

Understanding why and how extremely preterm infants die is important for practitioners caring for these infants.

**Objective:**

To examine risk factors, causes, timing, and circumstances of death in a modern cohort of extremely preterm infants.

**Design, Setting, and Participants:**

A retrospective cohort review of infants enrolled in the Preterm Erythropoietin Neuroprotection Trial between December 13, 2013, and September 26, 2016, was conducted. A total of 941 infants born between 24 0/7 and 27 6/7 weeks of gestation enrolled at 19 US sites comprising 30 neonatal intensive care units were included. Data analysis was performed from October 16, 2020, to December 1, 2021.

**Main Outcomes and Measures:**

Risk factors, proximal causes, timing, and circumstances of in-hospital death.

**Results:**

Of the 941 enrolled infants, 108 died (11%) before hospital discharge: 38% (n = 41) at 24 weeks’ gestation, 30% (n = 32) at 25 weeks’ gestation, 19% (n = 20) at 26 weeks’ gestation, and 14% (n = 15) at 27 weeks’ gestation. An additional 9 infants (1%) died following hospital discharge. In descending order, the primary causes of death included respiratory distress or failure, pulmonary hemorrhage, necrotizing enterocolitis, catastrophic intracranial hemorrhage, sepsis, and sudden unexplained death. Fifty percent of deaths occurred within the first 10 days after birth. The risk of death decreased with day of life and postmenstrual age such that an infant born at 24 weeks’ gestation who survived 14 days had the same risk of death as an infant born at 27 weeks’ gestation: conditional proportional risk of death, 0.08 (95% CI, 0.03-0.13) vs 0.06 (95% CI, 0.01-0.11). Preterm labor was associated with a decreased hazard of death (hazard ratio [HR], 0.45; 95% CI, 0.31-0.66). Infant clinical factors associated with death included birth weight below the tenth percentile for gestational age (HR, 2.11; 95% CI, 1.38-3.22), Apgar score less than 5 at 5 minutes (HR, 2.19; 95% CI, 1.48-3.24), sick appearance at birth (HR, 2.49; 95% CI, 1.69-3.67), grade 2b-3 necrotizing enterocolitis (HR, 7.41; 95% CI, 5.14-10.7), pulmonary hemorrhage (HR, 10.0; 95% CI, 6.76-18.8), severe intracranial hemorrhage (HR, 4.60; 95% CI, 3.24-5.63), and severe sepsis (HR, 4.93; 95% CI, 3.67-7.21). Fifty-one percent of the infants received comfort care before death.

**Conclusions and Relevance:**

In this cohort study, an association between mortality and gestational age at birth was noted; however, for each week that an infant survived, their risk of subsequent death approximated the risk observed in infants born 1 to 2 weeks later, suggesting the importance of an infant’s postmenstrual age. This information may be useful to include in counseling of families regarding prognosis of survival.

## Key Points

**Question:**

What are the risk factors, causes, timing, and circumstances of death for infants born at 24 0/7 to 27 6/7 weeks of gestation?

**Findings:**

In this cohort study of 941 infants enrolled in the Preterm Erythropoietin Neuroprotection Trial, mortality was lower and infants lived longer than in previous decades, and the most common cause of death was respiratory distress or failure. Survival increased with postnatal day and postmenstrual age; half of the dying infants received comfort care.

**Meaning:**

The findings of this study suggest that prognostication for extremely preterm infants should consider both gestational and postnatal age.

## Teaser

This cohort study examines the incidence and causes of death in US infants born extremely preterm.

## Introduction

More than 51 000 infants were born with a birth weight less than 1500 g in the US in 2019.^1^ Although the proportion of extremely preterm (EP) births has remained similar over the last quarter century,^2^ the age of viability has shifted substantially, attributable to ongoing advances in neonatal-perinatal medicine. The chances of survival at 24 to 25 weeks now range from 75% to 90%,^3,4^ compared with 62% to 68% in the late 1990s and early 2000s.^5,6^ However, changes in the cause and timing of death among these infants remain poorly described. Understanding modern trends relating to how and when EP infants die may provide valuable data to inform discussions with families regarding prognosis.

Important milestones in neonatology over the past 25 years include the standardization of antenatal corticosteroid administration, use of surfactant, caffeine, and more recently, increased use of noninvasive positive pressure support. Improvements with these therapies in both mortality and morbidity are well described,^7,8,9^ but how the timing, causes, and circumstances of death have changed remain unclear.

In this study, we used the robust database created by the Preterm Erythropoietin Neuroprotection (PENUT) Trial, which prospectively enrolled and randomized 941 EP infants at 19 sites, 30 neonatal intensive care units and 13 states across the US to evaluate infant and clinical risk factors for death and describe the circumstances and causes of death. Specifically, we investigated maternal and infant risk factors for death, the primary cause and timing of death, and how often death followed discontinuation of life-sustaining medical treatment in a modern cohort of EP infants born between 24 and 28 weeks’ gestation. Herein, we describe details associated with death among these infants and how key factors vary by week of gestation.

## Methods

### Data Source and Study Population

All infants born between December 13, 2013, and September 26, 2016, who were enrolled in the PENUT trial were eligible for this study. This included infants born between 24 0/7 and 27 6/7 weeks’ gestation without known life-threatening anomalies, chromosomal anomalies, disseminated intravascular coagulopathy, twin-to-twin transfusion, polycythemia, hydrops fetalis, or known congenital infection.^10^ The infant’s parents or legal guardian gave written informed consent for their participation in the PENUT trial. Details of the methods and primary outcome results have been published elsewhere.^10,11^ The PENUT trial was approved by the University Washington Institutional Review Board and by each recruiting site’s institutional review board. This study followed the Strengthening the Reporting of Observational Studies in Epidemiology (STROBE) reporting guideline for cohort studies.

Detailed demographic and clinical data were prospectively collected during the initial hospital stay because these details may be associated with the outcome of EP infants; data on race and ethnicity are required by the funding organization.^12^ Demographic information was reported by the mother. Infant information was collected prospectively.

Postnatal morbidities included the incidence and severity of common sequelae of prematurity: bronchopulmonary dysplasia (supplemental oxygen use at 36 weeks’ postmenstrual age [PMA]),^13^ any intracerebral hemorrhage (ICH) (Papile scoring method),^14^ periventricular leukomalacia,^15^ any culture-positive sepsis, stage 2 or greater necrotizing enterocolitis (NEC),^16^ patent ductus arteriosus, and any stage retinopathy of prematurity.^17^

Serious adverse events (SAEs) were defined a priori and included symptomatic thrombosis involving a major vessel unrelated to an infusion line and requiring anticoagulation (eg, superior vena cava syndrome), central hematocrit greater than 65% or hematocrit increase greater than or equal to 15% (to convert to proportion of 1.0, multiply by 0.01) in the absence of a preceding blood transfusion, hypertension (patient required antihypertensive therapy for more than 1 month and/or was discharged while receiving medication), severe pulmonary hemorrhage (associated with increased respiratory support), severe NEC (stage 2b or 3 Bell criteria),^16^ severe retinopathy of prematurity requiring laser surgery or bevacizumab therapy, severe sepsis (culture-proven bacterial or fungal sepsis requiring blood pressure support or significant new respiratory support), severe ICH (grade III/IV), cardiac arrest not resulting in death, other life-threatening event, or death.

### Deaths

A detailed description of the circumstances leading to death were prospectively recorded by study investigators and simultaneously adjudicated by an independent medical monitor. The cause of death was reviewed by one of us (S.E.J.) who then recorded the primary and secondary causes of death. Four infants who died before receiving study drug were excluded from the published modified intention-to-treat analysis of the primary outcome but are included here; 117 infants were lost to follow-up and no information about deaths was available for this subset. We explored whether there might be regional differences in the end-of-life decision-making process using 4 geographic regions, as defined in the National (Nationwide) Inpatient Sample^18^: West (New Mexico, Utah, and Washington), Midwest (Illinois and Minnesota), Northeast (Maryland, Massachusetts, and New York), and South (Arkansas, Florida, Kentucky, North Carolina, and Texas).

### Statistical Analysis

Data analysis was performed from October 16, 2020, to December 1, 2021. Simple descriptive statistics and graphic illustrations were used to initially describe the timing of in-hospital deaths stratified by gestational age (GA) and cause of death. For comparison with historical death data in a similar cohort, data were manually extracted from studies by Meadow et al^19^ and Patel et al.^9^ To compare survival by GA at birth and PMA, infants were aligned based on PMA and conditional future survival or subsequent survival from age = t was evaluated, defined as the percent of infants who survive to discharge among those who remained alive at age = t. Subsequent survival for different GA subgroups was then evaluated as P(discharge | GA = x, alive at age = t) = P(discharge | GA = x + t, alive at age = 0). For ultimate statistical inference, generalized estimating equations with robust SEs were used to appropriately account for potential correlation of outcomes for same-birth siblings.^20^ Using unadjusted generalized estimating equation logistic regression models as well as adjusted models taking into account GA and treatment, we estimated odds ratios (ORs) with 95% CIs to characterize the association between maternal and infant factors and occurrence of in-hospital death. For these risk factor comparisons, a Bonferroni correction was applied to the *P* value to adjust for multiple comparisons. The adjusted *P* value compared the nested model including the variable adjusted for gestational age and treatment group with a base model only including gestational and treatment group (with Bonferroni correction for 21 comparisons: adjusted significant *P* value = .05/21 = .0024). All other *P* values <.05 were considered statistically significant. Kaplan-Meier survival curves for survival in the first 10 weeks after birth were stratified by GA. For infant and postnatal SAEs, individual Cox proportional hazard ratio (HR) models were used to compare mortality risk over time, with SAEs treated as time-varying covariates. Additional Cox proportional hazard models were used to compare hazard of death by GA at birth and PMA in weeks, conditional on surviving to that PMA, as described above. All statistical analyses were performed using the R statistical software package, version 3.6.3 (R Foundation for Statistical Analysis), with additional figures produced using Prism, version 9.1 (Graphpad Software).

## Results

The Table reports the maternal demographic and clinical data comparing those who survived to discharge with those who died. Of the 941 enrolled infants, 108 died (11%) during the initial hospitalization, and 9 additional infants are known to have died following discharge. eFigure 1 in Supplement 1 shows a Consolidated Standards of Reporting Trials diagram, accounting for all infants screened and enrolled. Of the 108 in-hospital deaths, 38% (n = 41) occurred in infants born at 24 weeks’ gestation, 30% (n = 32) in infants born at 25 weeks’ gestation, 19% (n = 20) in infants born at 26 weeks’ gestation, and 14% (n = 15) in infants born at 27 weeks’ gestation. Of the 207 infants born at 24 weeks’ gestation, 21% died, compared with 16% of the 213 infants born at 25 weeks’ gestation, 10% of the 193 infants born at 26 weeks’ gestation, and 7% of the 206 infants born at 27 weeks’ gestation. Survival curves by gestational week are shown in eFigure 2 in Supplement 1. This figure shows the relatively steep curve for the most immature infants compared with those born at 27 weeks’ gestation. eFigure 3 in Supplement 1 shows the risk of subsequent death decreases with postnatal age such that the longer an infant survived, the higher the likelihood of subsequent survival. An infant’s PMA therefore appears to be the key time scale for characterizing risk of death. Specifically, we found that if an infant born at 24 weeks’ gestation survived 14 days, the likelihood of subsequent survival to discharge approximated the survival for those born at 27 weeks’ gestation. Similar age shifts in death risk took 10 days for an infant born at 25 weeks’ and 7 days for an infant born at 26 weeks’ gestation. Therefore, although these time shifts do not perfectly align with PMA time shifts (21 days for 26 weeks’, 14 days for 25 weeks’, and 7 days for 26 weeks’ gestation), it appears that prognosis should be updated from an initial description based on GA at birth to prognosis that focuses more on PMA as infants survive the initial high-risk period after birth. The risk of death decreased similarly for all groups thereafter. Conditional risk of death by week of PMA, stratified by GA at birth, is shown in eTable 1 in Supplement 1.

### Timing of Death

**Figure 1.**
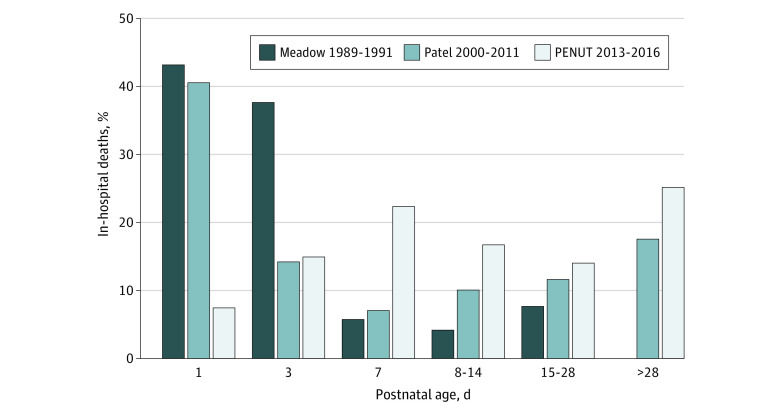
Percentage of Deaths as a Function of Postnatal Age The percentage of in-hospital deaths is shown by postnatal age for cohorts born 1989-1991 as reported by Meadow et al^19^ and 2000-2011 as reported by Patel et al,^9^ compared with the current 2013-2016 cohort from the Preterm Erythropoietin Neuroprotection (PENUT) trial. In the most current cohort, infants survived longer.

Of the 108 infants who died, 54 (50%) died in the first 10 days after birth: 28 (26%) died by the end of day 3, 40 (37%) died between 4 and 14 days of age, and 38 (35%) died after day 14.^19^ Figure 1 shows timing of death in 3 cohorts: 400- to 1000-g infants admitted to the neonatal intensive care unit between 1989 and 1991 (n = 227),^19^ liveborn infants 22 0/7 to 28 6/7 weeks’ gestation born between 2000 and 2011 (n = 6075),^9^ and the PENUT cohort, with parental consent for study given by 24 hours of age between 2013 and 2016.^10^ There was a marked shift in the timing of death over these epochs, with deaths occurring at a later postnatal age in the most recent epoch. In the first epoch, over 80% of deaths occurred in the first 3 days; in the second, 78% occurred by day 7; and in the current cohort, 80% died within the first 41 days.

### Risk Factors for Death

**Figure 2.**
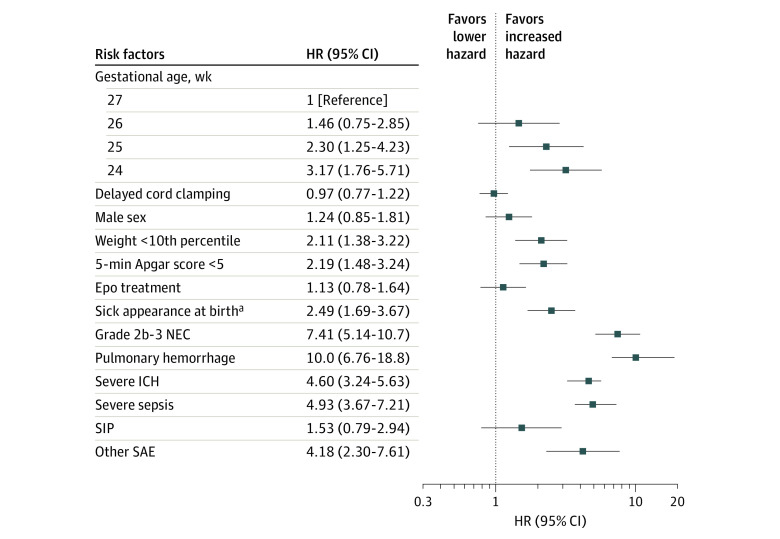
Infant Factors and Serious Adverse Events (SAEs) Associated With Death Hazard ratios (HRs) for in-hospital death by infant risk factors and common SAEs. Each factor was considered separately, so the HR for each factor represents the result of a separate Cox proportional hazard model. All models were adjusted for treatment group and gestational age at birth. SAEs were assessed as time-varying covariates for risk of death, with the HR adjudicated from the time the SAE occurred. Epo indicates erythropoietin; ICH, intracerebral hemorrhage; NEC, necrotizing enterocolitis; and SIP, spontaneous intestinal perforation. ^a^Sick appearance at birth was a subjective assessment by the attending neonatologist.

Self-reported maternal ethnicity, race, education, and prenatal care did not substantially associate with risk of infant death, nor did maternal drug, tobacco, or alcohol use (Table). Maternal conditions, such as preeclampsia, obesity, gestational diabetes, prolonged rupture of membranes, delivery complications, or chorioamnionitis, also did not appear to increase the risk of infant death. Accounting for multiple comparisons, preterm labor significantly decreased the risk of death (OR, 0.91; 95% CI, 0.87-0.96; *P* < .001), and receipt of at least 1 dose of prenatal corticosteroids was suggestive of a decreased risk (OR, 0.90; 95% CI, 0.82-0.99; *P* = .03). Figure 2 shows relevant infant risk factors and their association with the hazard of death. Factors that significantly increased the hazard of death included gestational age, weight below the 10th percentile for GA (HR, 2.11; 95% CI, 1.38-3.22), an Apgar score of less than 5 at 5 minutes (HR, 2.19; 95% CI, 1.48-3.24), an assessment of sick appearance at birth by the attending neonatologist, and all predesignated SAEs. Overall survival and HRs were evaluated according to whether infants developed SAEs. Among infants who did not develop severe sepsis, severe ICH, severe pulmonary hemorrhage, or NEC, only 32 infants (9%) died before discharge. When looking at SAEs individually, of the 65 infants who developed NEC, 28 died (43%) (HR, 7.41; 95% CI, 5.14-10.7). Of the 50 infants who developed a serious pulmonary hemorrhage, 26 died (52%) (HR, 10.0; 95% CI, 6.76-18.8). Of the 123 infants with a grade III/IV hemorrhage recorded, 32 died (26%) (HR, 4.60; 95% CI, 3.24-5.63). Of the 78 infants who developed severe sepsis, 27 died (35%) (HR, 4.93; 95% CI, 3.67-7.21). Of the 42 infants who developed spontaneous intestinal perforation, 6 died (14%) (HR, 1.53; 95% CI, 0.79-2.94). Serious adverse events coded as other included pulmonary hypertension (n = 5), meningitis/ventriculitis (n = 5), respiratory failure/bronchopulmonary dysplasia (n = 3), cerebellar hemorrhage (n = 2), and bowel obstruction (n = 2), and single reports of pneumopericardium, pulmonary valve stenosis, and hydrocephalus of unknown origin. The 6 deaths that occurred in infants with other SAEs (26%) (HR, 4.18; 95% CI, 2.30-7.61) included pneumopericardium (n = 1), pulmonary hypertension (n = 4), meningitis (n = 1), and liver failure (n = 1).

**Table.** Maternal Demographic and Clinical Characteristics

Maternal data	No. (%)		*P* value^a^	Adjusted *P* value^a^^,^^b^
	Survived	Died		
No. (%)	830 (88.5)	108 (11.5)	NA	NA
Age, mean (SD), y	28.8 (6.1)	30.5 (6.5)	.03	.01
Ethnicity				
Hispanic	174 (87.0)	26 (13.0)	Reference	.46
Not Hispanic	645 (88.8)	81 (11.2)	.49	
Unknown/not reported	11 (91.7)	1 (8.3)	NA	NA
Race^c^				
White	536 (87.3)	78 (12.7)	1 [Reference]	.14
African American/Black	214 (89.9)	24 (10.1)	.32	
American Indian/Alaska Native	15 (93.8)	1 (6.2)	NA	NA
Asian	26 (92.9)	2 (7.1)	NA	NA
Native Hawaiian or Other Pacific Islander	9 (100)	0	NA	NA
White/other	3 (100)	0	NA	NA
Unknown/not reported	27 (90.0)	3 (10.0)	NA	NA
Educational level				
High school or less	277 (90.2)	30 (9.8)	1 [Reference]	.21
Some college	254 (89.4)	30 (10.6)	.88	
College degree or greater	207 (88.5)	27 (11.5)	.68	
Not reported	92 (81.4)	21 (18.6)	.04	
Prenatal care				
No	24 (85.7)	4 (14.3)	1 [Reference]	.86
Yes	791 (88.5)	103 (11.5)	.83	
Not reported	15 (93.8)	1 (6.3)	NA	NA
Gravida, median (IQR)	2 (1-4)	2 (1-4)	.59	.62
Parity, median (IQR)	1 (0-2)	1 (0-2)	.48	.47
Recreational drug use				
No	732 (88.7)	93 (11.3)	1 [Reference]	.97
Yes	55 (88.7)	7 (11.3)	.95	
Not reported	43 (86.0)	7 (14.0)	NA	NA
Prescription drugs during pregnancy				
No	510 (88.9)	64 (11.1)	1 [Reference]	.73
Yes	259 (87.5)	37 (12.5)	.64	
Not reported	51 (89.5)	6 (10.5)	NA	NA
Tobacco use during pregnancy				
No	677 (88.6)	87 (11.4)	1 [Reference]	.59
Yes	101 (90.2)	11 (9.8)	.59	
Not reported	52 (83.9)	10 (16.1)	NA	NA
Alcohol use during pregnancy				
No	761 (88.8)	96 (11.2)	1 [Reference]	.84
Yes	17 (85.0)	3 (15.0)	.90	
Not reported	52 (85.2)	9 (14.8)	NA	NA
**Maternal complications**				
Eclampsia				
No	820 (88.6)	106 (11.4)	NA	NA
Yes	10 (83.3)	2 (16.7)		
Thyroid disease				
No	795 (88.2)	106 (11.8)	NA	NA
Yes	35 (94.6)	2 (5.4)		
Obesity				
No	746 (88.3)	99 (1.7)	1 [Reference]	.60
Yes	84 (90.3)	9 (9.7)	.62	
Ruptured membranes >18 h				
No	608 (88.5)	79 (11.5)	1 [Reference]	.95
Yes	222 (88.4)	29 (11.6)	.96	
Chorioamnionitis (suspected or confirmed)				
No	720 (88.3)	95 (11.7)	1 [Reference]	.59
Yes	110 (89.4)	13 (10.6)	.75	
Gestational diabetes				
No	785 (88.4)	103 (11.6)	1 [Reference]	.97
Yes	45 (90.0)	5 (10.0)	.87	
Preterm labor				
No	301 (83.4)	60 (16.6)	1 [Reference]	<.001
Yes	529 (91.7)	48 (8.5)	<.001	
Pyrexia, temperature >38 °C				
No	811 (88.4)	106 (11.6)	NA	NA
Yes	19 (90.5)	2 (9.5)		
Antibiotic administration				
No	528 (88.1)	71 (11.9)	1 [Reference]	.42
Yes	302 (89.1)	37 (10.9)	.64	
Pregnancy-induced hypertension				
No	769 (88.7)	98 (11.3)	1 [Reference]	.40
Yes	61 (85.9)	10 (14.1)	.46	
Prenatal corticosteroids				
No	62 (79.5)	16 (20.5)	1 [Reference]	.03
Yes	755 (89.6)	88 (10.4)	.04	
Unknown/not reported	13 (76.5)	4 (23.5)	NA	NA
Prenatal corticosteroids (≥2 doses)				
No	158 (90.3)	17 (9.7)	1 [Reference]	.53
Yes	589 (89.2)	71 (10.8)	.62	
Unknown/not reported	83 (80.6)	20 (19.4)	NA	NA
Prenatal magnesium sulfate				
No	128 (86.5)	20 (13.5)	1 [Reference]	.49
Yes	669 (89.1)	82 (10.9)	.40	
Unknown/not reported	32 (84.2)	6 (15.8)	NA	NA
Delivery complications				
No	697 (88.6)	90 (11.4)	1 [Reference]	.98
Yes	133 (88.1)	18 (11.9)	.90	
Cesarean delivery				
No	260 (90.6)	27 (9.4)	1 [Reference]	.10
Yes				
Elective	72 (92.3)	6 (7.7)	.87	
Urgent/emergency	498 (86.9)	75 (13.1)	.07	
Multiple gestation				
No	612 (89.1)	75 (10.9)	1 [Reference]	.36
Yes	218 (86.9)	33 (13.1)	.39	

^a^ *P* values not calculated for factors when variables were unknown, not reported, or the prevalence of a category was under 5% of the number of infants.

^b^ Adjusted *P* value compared nested model including the variable adjusted for gestational age and treatment group with a base model only including gestational and treatment group (with Bonferroni correction for 21 comparisons: adjusted significant *P* value = .05/21 = .0024.

^c^ White/Other category determined by those who selected the option “plus another category” (n = 2) and American Indian/Alaska Native (n = 1).

### Cause of Death

**Figure 3.**
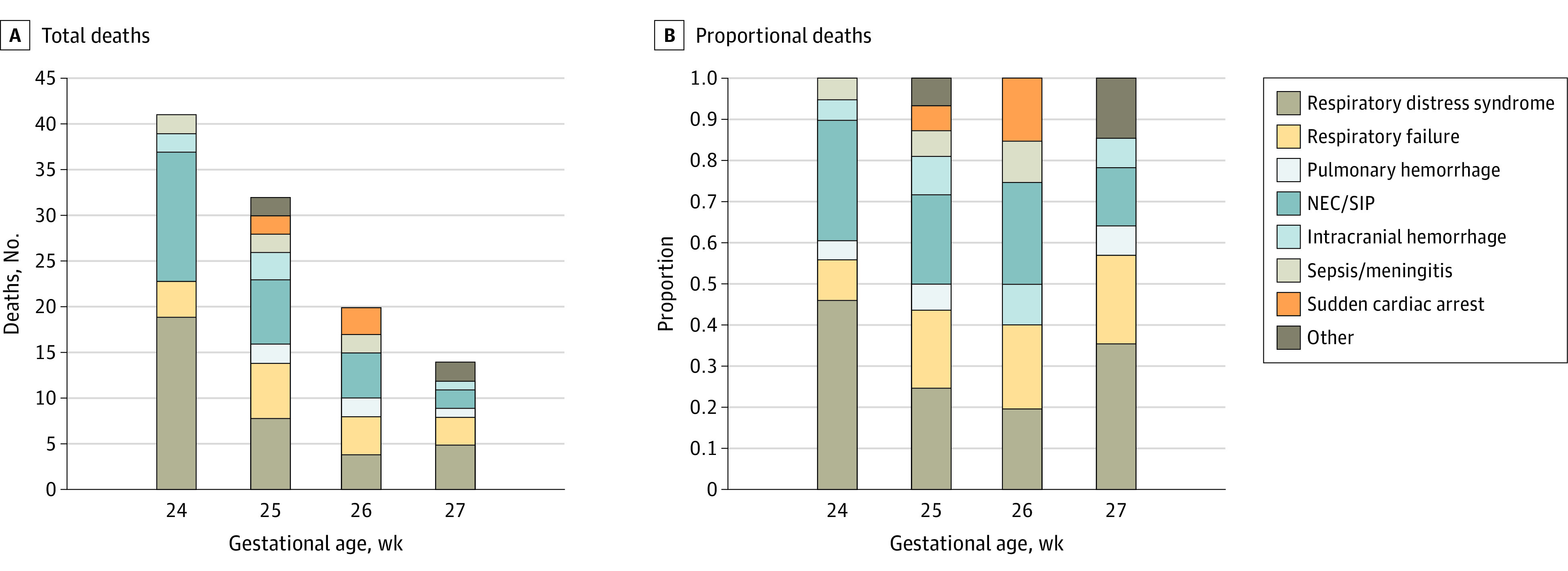
Cause of Death by Gestational Age Total deaths (A) and normalized proportion of deaths (B) by gestational age, with proximal cause of death shown. NEC indicates necrotizing enterocolitis; SIP, spontaneous intestinal perforation.

**Figure 4.**
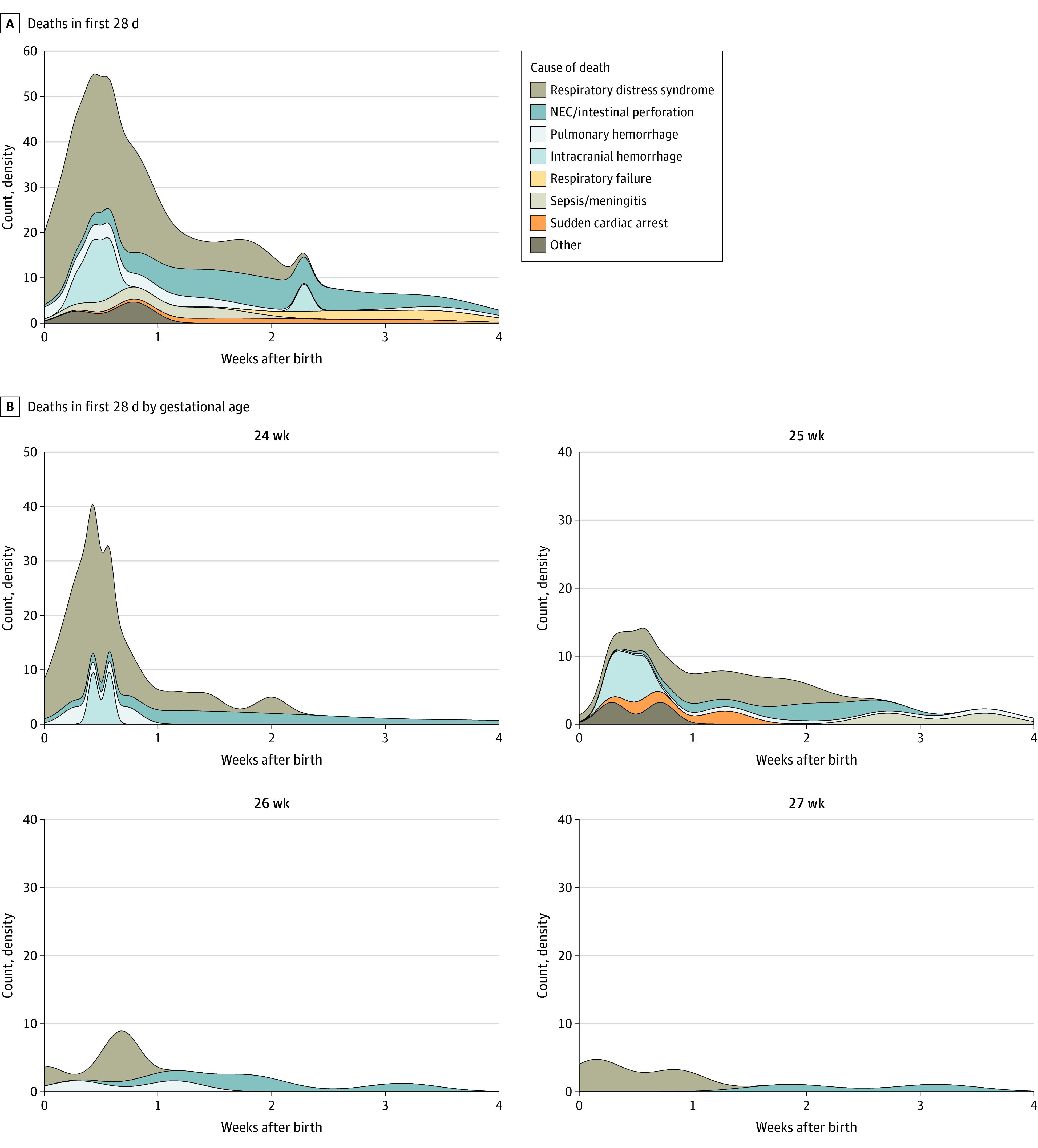
In-Hospital Deaths by Cause in the Neonatal Period Timing and proximal cause of death for all neonatal deaths (first 28 days of life) (A) and separated by week of gestation (B). The total number of in-hospital neonatal deaths for each cause of death were respiratory distress syndrome, n = 36; necrotizing enterocolitis (NEC)/intestinal perforation, n = 17; pulmonary hemorrhage, n = 7; intracranial hemorrhage, n = 6; respiratory failure, n = 4; sepsis/meningitis, n = 4; sudden cardiac arrest, n = 4; and other, n = 3.

The primary causes of death in order of frequency included respiratory distress syndrome, NEC, spontaneous intestinal perforation, late respiratory failure (defined as ongoing respiratory compromise after 2 weeks of life), pulmonary hemorrhage, sepsis/meningitis, catastrophic ICH, and sudden death of undetermined cause. Figure 3A shows the number of infants who died by gestational week and their causes of death; Figure 3B shows the normalized causes of death. The combination of NEC/spontaneous intestinal perforation and respiratory causes of death accounted for 85% of deaths at 24 weeks, 66% at 25 weeks, 65% at 26 weeks, and 71% at 27 weeks. Figure 4 shows the timing and cause of death for all infants in the first 28 days after birth. Respiratory causes (respiratory distress syndrome, pulmonary hemorrhage, and late respiratory failure) made up more than half of all deaths. Of the 108 in-hospital deaths, 81 (75%) occurred in the first 28 days, including 32 (78%) in those born at 24’ weeks gestation, 23 (78%) born at 25’ weeks gestation, 13 (65%) born at 26’ weeks gestation, and 11 (73%) born at 27 weeks’ gestation (Figure 3B). In the 24-week gestation group, a higher proportion of deaths occurred early. eFigure 4 in Supplement 1 is a swimmer plot showing the medical course and cause of death for each infant within the neonatal period (eFigure 4A in Supplement 1) or after 4 weeks (eFigure 4B in Supplement 1), with the timing and nature of SAEs designated as whether life-sustaining care was withdrawn. Necrotizing enterocolitis was the proximal cause of death for 30% of infants born at 24 weeks’ gestation, 22% of infants born at 25 weeks’ gestation, 25% of infants born at 26 weeks’ gestation, and 14% of infants born at 24 weeks’ gestation. Late in hospital deaths were most commonly due to late respiratory failure, complications of NEC, or sudden cardiac arrest. These infants often had ongoing multisystem organ failure. Of the 9 children (age, 6-24 months’ corrected age) who died after discharge, 3 died suddenly and unexpectedly and 6 died of multiorgan failure.

Discontinuation of life-sustaining medical treatment ranged from 0 to 100% by site, with a median percentage of 40% (IQR, 33%-75%) of deaths that included withdrawal of care. Withdrawal was documented in 42% of infants who died of respiratory distress syndrome, 69% of those who died with NEC, 79% of those with respiratory failure, 14% of those who died with pulmonary hemorrhage or sepsis/meningitis, and 83% of those who died due to intracranial hemorrhage. Discontinuation of life-sustaining medical treatment most often occurred within 1 hour or 2 hours before death to facilitate parents holding of their critically ill infant. Overall, 51% of these extremely preterm infants were extubated and held as they died. eTable 2 in Supplement 1 reports the demographic information defined by withdrawal status (yes vs no). By region, 56% (n = 15/27) of the deaths in the Midwest, 57% (n = 8/14) in the West, 53% (n = 24/45) in the South, and 33% (n = 7/21) in the Northeast region involved discontinuation of life-sustaining care. All PENUT sites were referral centers located in counties politically considered majority Democratic, although 9 of the 17 sites were in states considered Republican. When viewed by state, the neonatal intensive care units in Democratically-leaning states had a withdrawal rate of 52% of deaths, while in the Republican-leaning states, withdrawal rate was 46%. No local or maternal demographic variables were significantly associated with the likelihood of withdrawing life-sustaining care.

## Discussion

We have examined details of the causes and timing of deaths in a recent cohort of infants born at 24 to 27 completed weeks of gestation. Overall mortality was lower than expected, at 12% within the initial hospitalization, with an additional 1% who died between 9 and 25 months after birth.^9,21,22,23^ As expected, the risk of death was highest in the least mature infants; however, the ability of GA to estimate outcome decreased after 2 postnatal weeks such that a 2-week-old infant born at 24 weeks’ gestation had the same risk of death as a newborn infant born at 27 weeks’ gestation. The interval between birth and death has lengthened over the past decades, reflecting advances in both perinatal and neonatal care. In 1996, over 80% of deaths occurred in the first 3 days,^19^ in 2000-2011, 54% of deaths occurred in the first 3 days,^9^ whereas in the present cohort, only 21% of deaths had occurred by 3 days after birth, and it took 41 days for 80% to have died. The PENUT trial only included infants who survived to be enrolled, so this difference is accentuated. Mortality was also lower and time to death longer in our cohort than reported in a recent cohort from the Netherlands.^22^ Mortality details have implications for parental guidance: in 1996 one could tell parents that if their child survived 3 days, the chances for ongoing survival were high, whereas in our present cohort, the period of uncertainty regarding ultimate outcome is prolonged and has the potential to increase parental stress.

Respiratory causes remain the most prevalent cause of death, with respiratory distress syndrome, pulmonary hemorrhage, and late respiratory failure being the cause of death for between 50% and 60% of all deaths. Necrotizing enterocolitis decreased as a proximal cause of death with increasing maturity, ranging from 30% to 14% as gestational age at birth increased from 24 to 27 weeks’ gestation. Catastrophic ICH was less common as a cause of death, ranging from 0 to 9% across all GA categories.

Half of the infants who died in this cohort had life-sustaining care withdrawn, most often when the inevitability of death was certain, and the child died within an hour or 2. Withdrawal was done primarily so the child could be held and die peacefully in the parent’s arms. In previous studies, withdrawal of life-sustaining care was more often done for quality-of-life issues and differed by region of the country.^24,25^ There was no significant difference in withdrawal of care by maternal demographics or region of the country, although the Northeastern sites have had lower rates in withdrawal of care.

### Limitations

The study has limitations. Our study involved a multicenter population in the US. In addition, the PENUT centers were referral centers, and primarily academic; thus, deaths of EP infants cared for at smaller community hospitals are not considered. In addition, infants whose parents did not consent to PENUT, those who were excluded or died before enrollment in PENUT, and infants born at 22 and 23 weeks’ gestation were not included. Additional children who were lost to follow-up by the end of the PENUT trial (22-26 months’ corrected age) may have died. Resuscitation of infants less than 24 weeks’ gestation is becoming increasingly common, with survival rates improving with practice change.^26^ The timing and causes of death for these infants is not well studied so we cannot comment on whether their deaths follow a similar pattern as the infants described in this study.

## Conclusions

Mortality among EP infants continues to decrease over time. Contemporary data on the timing, causes, and factors associated with death provide the critical summary information that is necessary for clear and accurate counseling of parents and caregivers.

## Notes

Supplement 1. **eFigure 1.** CONSORT Diagram **eFigure 2.** Survival Curves per Completed Week of Gestation **eFigure 3.** Risk of Death Over Time by Gestational Age at Birth **eFigure 4.** Hospital Course for Each Child Including SEAs and Timing of Death **eTable 1.** Conditional Risk of Death through Discharge **eTable 2.** Withdrawal of Life-Sustaining Care by Demographics

Supplement 2. **Nonauthor Collaborators.** The PENUT Consortium
