# Supplementary material for: Deaths in a Modern Cohort of Extremely Preterm Infants From the Preterm Erythropoietin Neuroprotection Trial
Source: JAMA Netw Open. 2022 Feb 7;5(2):e2146404. doi: 10.1001/jamanetworkopen.2021.46404 (PMC8822378; doi:10.1001/jamanetworkopen.2021.46404)
Supplement: Supplement 1. — eFigure 1. CONSORT Diagram eFigure 2. Survival Curves per Completed Week of Gestation eFigure 3. Risk of Death Over Time by Gestational Age at Birth eFigure 4. Hospital Course for Each Child Including SEAs and Timing of Death eTable 1. Conditional Risk of Death through Discharge eTable 2. Withdrawal of Life-Sustaining Care by Demographics [file jamanetwopen-e2146404-s001.pdf]

Supplementary Online Content

Juul SE, Wood TR, Comstock BA, et al; PENUT Consortium. Deaths in a modern cohort of extremely preterm infants from the Preterm Erythropoietin Neuroprotection Trial. *JAMA Netw Open*. 2022;5(2):e2146404. doi:10.1001/jamanetworkopen.2021.46404

eFigure 1. CONSORT Diagram

eFigure 2. Survival Curves per Completed Week of Gestation

eFigure 3. Risk of Death Over Time by Gestational Age at Birth

eFigure 4. Hospital Course for Each Child Including SEAs and Timing of Death

eTable 1. Conditional Risk of Death through Discharge

eTable 2. Withdrawal of Life-Sustaining Care by Demographics

This supplementary material has been provided by the authors to give readers additional information about their work.

© 2022 Juul SE et al. *JAMA Network Open.*

eFigure 1. CONSORT diagram

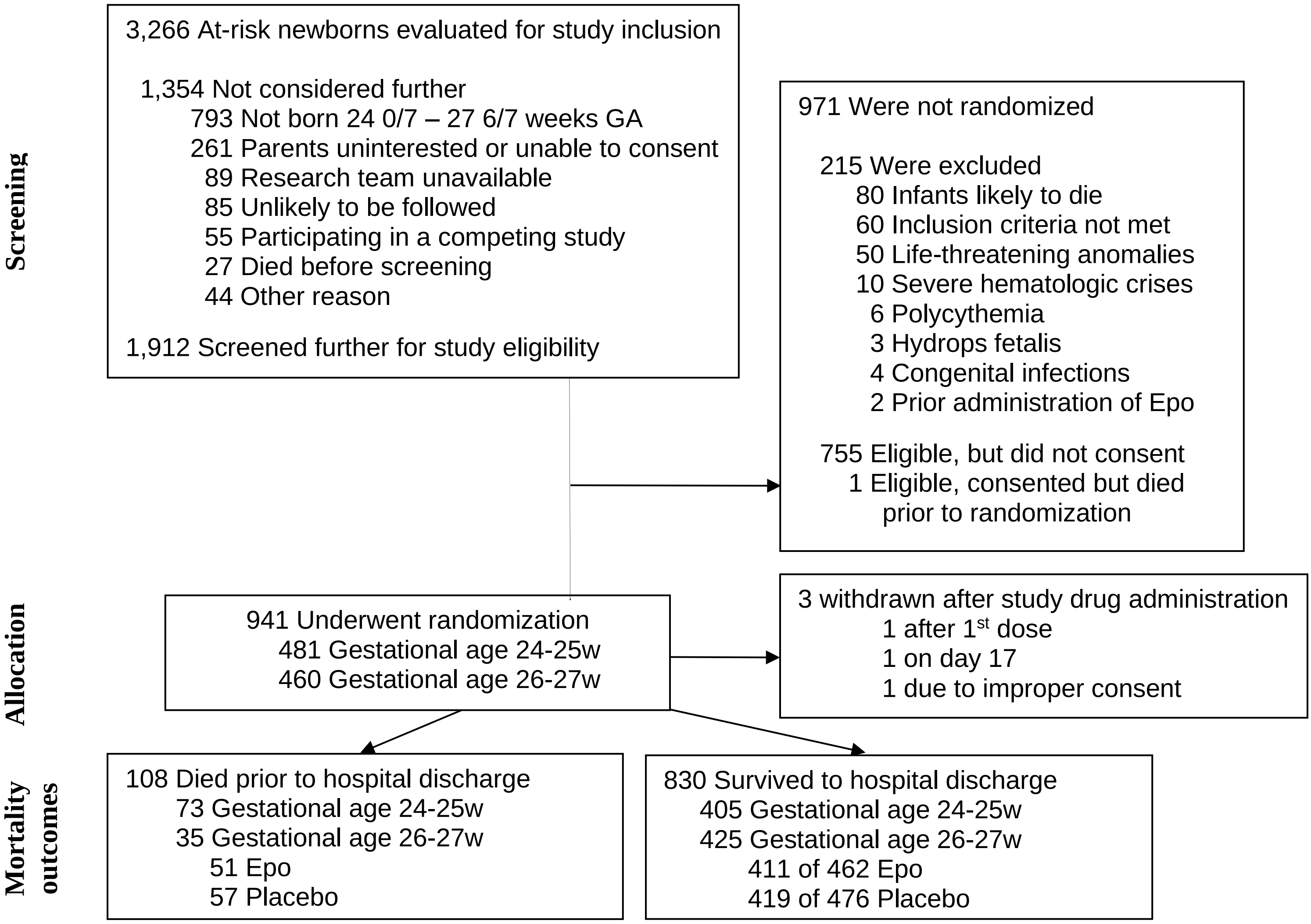

eFigure 1. The CONSORT diagram shows all infants screened, enrolled and randomized to treatment groups in the PENUT Trial, including those who survived or died prior to hospital discharge. Nine children are known to have died following discharge.

© 2022 Juul SE et al. *JAMA Network Open.*

eFigure 2. Survival Curves per Completed Week of Gestation

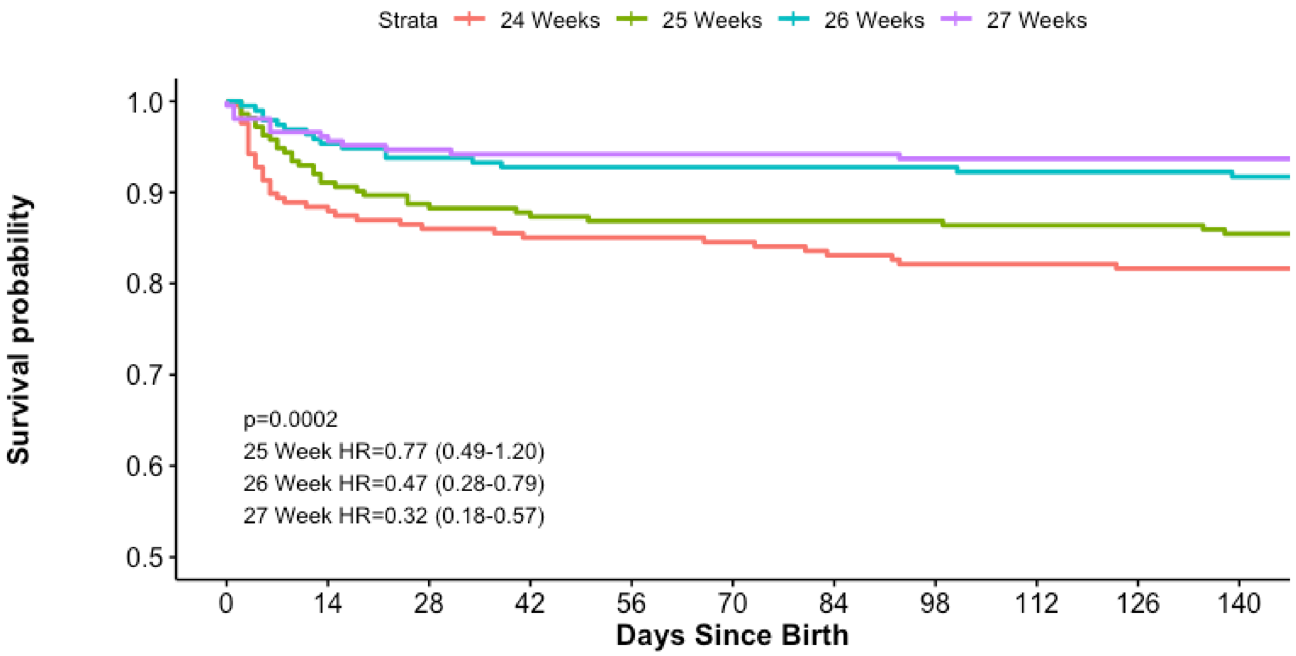

Number at risk

	0	14	28	42	56	70	84	98	112	126	140
24 Weeks	207	183	178	176	176	175	172	170	170	169	169
25 Weeks	213	194	189	187	185	185	185	185	184	184	182
26 Weeks	193	184	181	179	179	179	179	179	178	178	177
27 Weeks	206	198	195	194	194	194	194	193	193	193	193

eFigure 2.

Kaplan-Meier curve showing survival curves by gestational age for the first 20 weeks after birth. The accompanying table gives the number of surviving infants at each time interval.

© 2022 Juul SE et al. *JAMA Network Open.*

eFigure 3. **Risk of Death Over Time by Gestational Age at Birth**

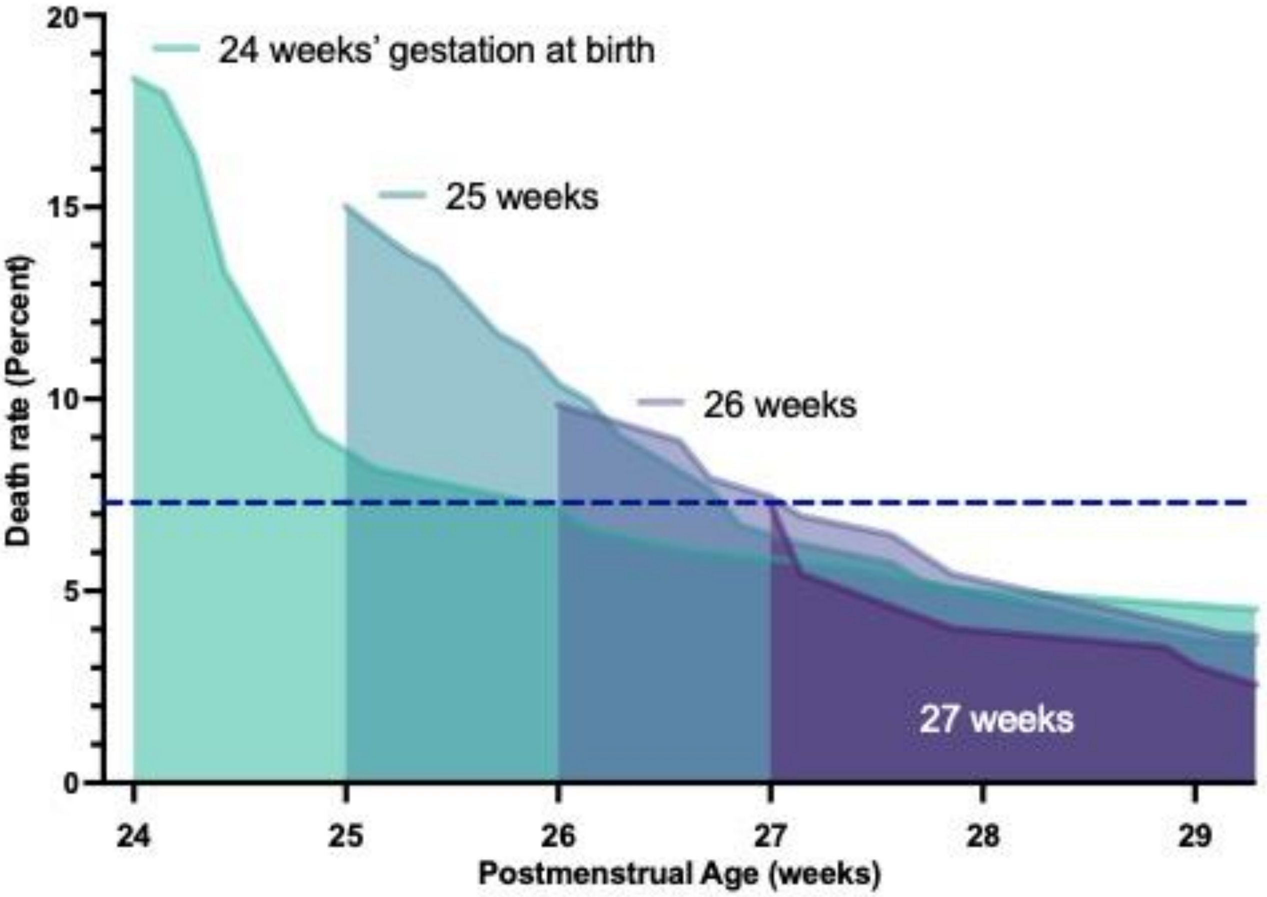

eFigure 3:
The rate and timing of in-hospital death for infants born at each week of gestation is shown. The dotted horizontal line shows the risk of death for those infants born at 27 weeks. The point at which each curve crosses the dotted line shows when the risk of death for that group is the same as for infants born at 27 weeks of gestation. For instance, an infant born at 24 weeks' gestation reaches this risk strata after 14 days, while an infant born at 25 weeks reaches this after 10 days, and an infant born at 26 weeks reaches this after 7 days. Thus, both gestational age at birth and postmenstrual age affect the risk of death.

© 2022 Juul SE et al. *JAMA Network Open.*

eFigure 4. Hospital course for each child including SAEs and timing of death.

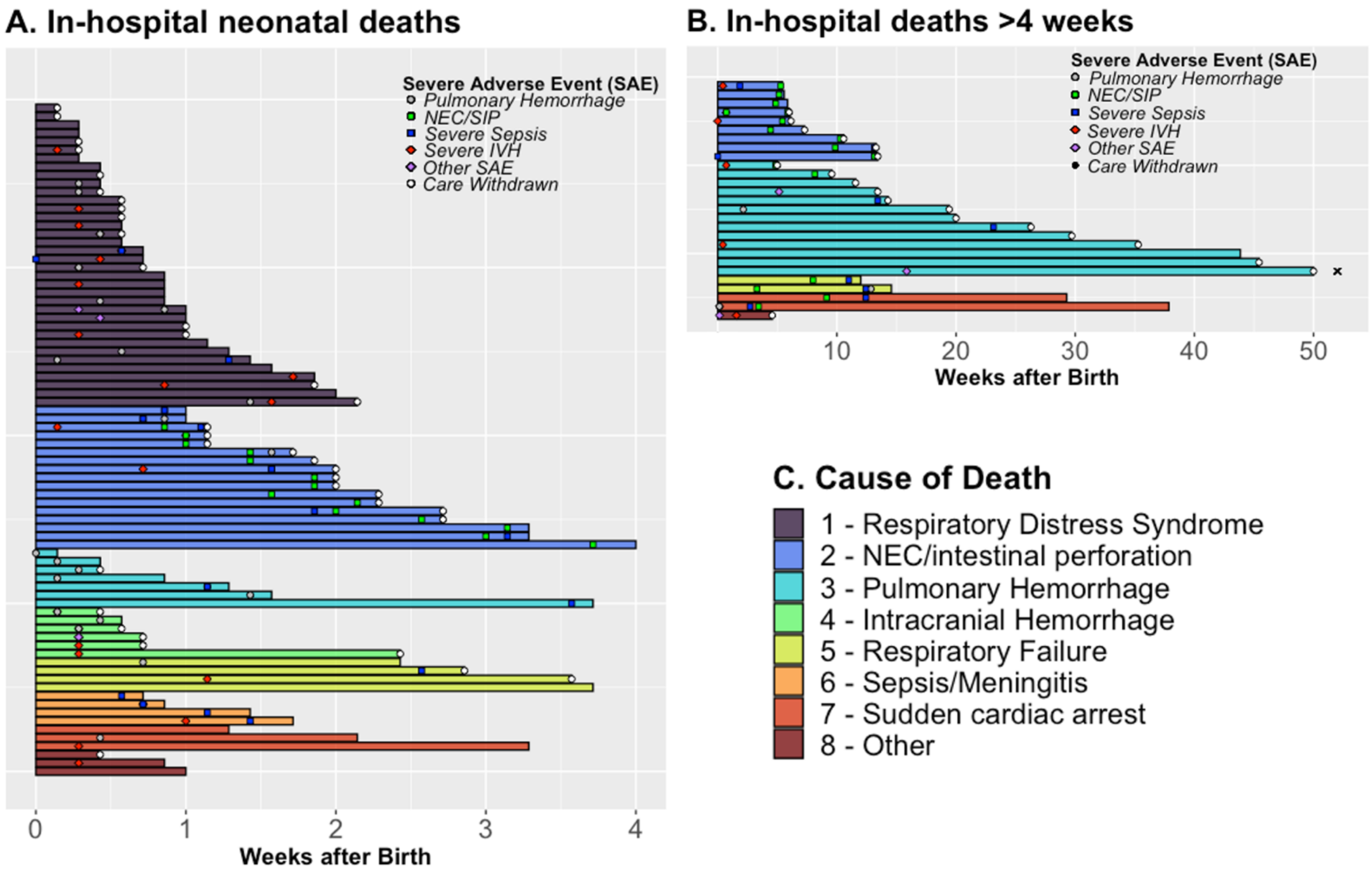

eFigure 4. Swimmer plots showing timing of in-hospital death and common SAEs, with deaths grouped by cause in the neonatal period (A) and over the entire hospital course (B). One infant who died before discharge at 90 weeks was censored at 50 weeks, denoted by an x. Each horizontal line represents one child, with the SAEs they experienced, the timing of death, and whether care was withdrawn prior to death.

© 2022 Juul SE et al. *JAMA Network Open.*

eTable 1. Conditional Risk of Death through Discharge. Conditional risk of death (proportion of infants who die before discharge with 95% CI) at each week of postmenstrual age (PMA), stratified by gestational age (GA) at birth. Each proportion is calculated conditional on infants surviving to that PMA, where: P(death-by-discharge | GA=x, survival to PMA=t).

	**Conditional Risk of Death through Discharge (95% CI) by Postmenstrual Age**					
	24 Weeks	25 Weeks	26 Weeks	27 Weeks	28 Weeks	29 Weeks
**24 Weeks GA**	0.19 (0.12-0.26)	0.09 (0.04-0.15)	0.08 (0.03-0.13)	0.07 (0.02-0.12)	0.06 (0.01-0.10)	0.06 (0.01-0.10)
**25 Weeks GA**		0.11 (0.05-0.16)	0.08 (0.03-0.13)	0.04 (0.00-0.07)	0.03 (0.00-0.06)	0.03 (0.00-0.06)
**26 Weeks GA**			0.08 (0.03-0.14)	0.07 (0.02-0.11)	0.05 (0.01-0.09)	0.05 (0.01-0.09)
**27 Weeks GA**				0.06 (0.01-0.11)	0.04 (0.00-0.08)	0.04 (0.00-0.08)
p-value*	-	0.27	0.31	0.43	0.43	0.43

*P-value compares a Cox proportional hazards model at that PMA adjusting for treatment group only to a model also including GA at birth, conditional on surviving to that PMA.

© 2022 Juul SE et al. *JAMA Network Open.*

eTable2. Withdrawal of Life-Sustaining Care by Demographics			
	Total<br>n (%)	Died<br>n (%)	Died after withdrawal of life-sustaining care<br>n (% of those that died)
	941	108 (11.5%)	53 (49.1%)
**Maternal Data**			
**Age, mean (SD)**	29.0 (6.2)	30.5 (6.5)	29.9 (6.0)
**Ethnicity**			
Hispanic	200	26 (13%)	11 (42.3%)
Not Hispanic	728	81 (11.1%)	41 (50.6%)
Unknown/Not Reported	12	1 (8.3%)	1 (100%)
**Race***			
African American/Black	238	24 (10.1%)	15 (62.5%)
Asian	31	2 (6.5%)	2 (100%)
Native American/Alaskan	16	1 (6.3%)	0 (0%)
Native Hawaiian/Pacific Islander	9	0 (0%)	-
White	614	78 (12.7%)	35 (44.9%)
Unknown/Not Reported	30	3 (10.0%)	2 (66.7%)
**Education**			
High School or less	307	30 (9.8%)	15 (50.0%)
Some college	284	30 (10.6%)	15 (50.0%)
College degree or greater	234	27 (11.5%)	13 (48.1%)
Not Reported	113	21 (18.6%)	10 (47.6%)

Percentages are row percentages within a given demographic level.

© 2022 Juul SE et al. *JAMA Network Open.*
