# Supplementary material for: Deaths in a Modern Cohort of Extremely Preterm Infants From the Preterm Erythropoietin Neuroprotection Trial
Source: JAMA Netw Open. 2022 Feb 7;5(2):e2146404. doi: 10.1001/jamanetworkopen.2021.46404 (PMC8822378; doi:10.1001/jamanetworkopen.2021.46404)
Supplement: Supplement 2. — Nonauthor Collaborators. The PENUT Consortium [file jamanetwopen-e2146404-s002.pdf]

*Indicates required information. Only first name, last name, and suffix will appear in PubMed.

*Group Name(s): PENUT Consortium							
***First Name and Middle Initial(s)**	***Last Name**	***Suffix (eg, Jr, III)**	Academic Degrees	Institution	Location (city, state/province, country)	Role or Contribution, eg, chair, principal investigator	Group (if more than 1 Group listed in the byline) and/or Subgroup (eg, Steering Committee)
Kaashif A.	Ahmad		MD, MSc	Methodist Children's Hospital	San Antonio, Texas	PENUT Site PI	
Mariana	Baserga		MD	University of Utah	Salt Lake City, Utah	PENUT Site PI	
Ellen	Bendel-Stenzel		MD	Children's Minnesota	Minneapolis, Minnesota	PENUT Site PI	
Sherry E.	Courtney		MD	University of Arkansas for Medical Sciences	Little Rock, Arkansas	PENUT Site PI	
L. Corbin	Downey		MD	Wake Forest School of Medicine	Winston-Salem, North Carolina	PENUT Site PI	
Nancy	Fahim		MD, MSc	University of Minnesota Masonic Children's Hospital	Minneapolis, Minnesota	PENUT Site PI	
Ivan D.	Frantz	III	MD	Beth Israel Deaconess Medical Center	Boston, Massachusetts	PENUT Site PI	
Maureen M.	Gilmore		MD	Johns Hopkins University	Baltimore, Maryland	PENUT Site PI	
Janine	Khan		MD	Prentice Women's Hospital	Chicago, Illinois	PENUT Site PI	
Edmund F.	LaGamma		MD	Maria Fareri Children's Hospital at Westchester Medical Center	Valhalla, New York	PENUT Site PI	
Andrea	Lampland		MD	Children's Minnesota	St. Paul, Minnesota	PENUT Site PI	
Jean	Lowe		PhD	University of New Mexico	Albuquerque, New Mexico	PENUT Site PI	
Dennis E.	Mayock		MD	University of Washington	Seattle, Washington	PENUT Site PI	PENUT Executive Committee
Victor	McKay		MD	Johns Hopkins All Children's Hospital	St. Petersburg, Florida	PENUT Site PI	
Robin K.	Ohls		MD	University of New Mexico	Albuquerque, New Mexico	PENUT Site PI	
Jorge E.	Perez		MD	South Miami Hospital	South Miami, Florida	PENUT Site PI	

Supplemental Online Content: Nonauthor Collaborators

*Indicates required information. Only first name, last name, and suffix will appear in PubMed.

***First Name and Middle Initial(s)**	***Last Name**	***Suffix (eg, Jr, III)**	Academic Degrees	Institution	Location (city, state/province, country)	Role or Contribution, eg, chair, principal investigator	Group (if more than 1 Group listed in the byline) and/or Subgroup (eg, Steering Committee)
Raghavendra	Rao		MD	University of Minnesota Masonic Children's Hospital	Minneapolis, Minnesota	PENUT Site PI	
Tonya	Robinson		MD	University of Louisville	Louisville, Kentucky	PENUT Site PI	
Nishant	Srinivasan		MD	Children's Hospital of the University of Illinois	Chicago, Illinois	PENUT Site PI	
Rajan	Wadhawan		MD	Advent Health for Children	Orlando, Florida	PENUT Site PI	
Michael	Weiss		MD	University of Florida	Gainesville, Florida	PENUT Site PI	
Billy	Thomas		MD, MPH	University of Arkansas for Medical Sciences	Little Rock, Arkansas	PENUT Site Co-I	
Nahed	Elhassan		MD, MPH	University of Arkansas for Medical Sciences	Little Rock, Arkansas	PENUT Site Co-I	
Sarah	Mulkey		MD, PhD	University of Arkansas for Medical Sciences	Little Rock, Arkansas	PENUT Site Co-I	
Vivek K.	Vijayamadhavan		MD	Methodist Children's Hospital	San Antonio, Texas	PENUT Site Co-I	
Neil	Mulrooney		MD	Children's Minnesota	Minneapolis, Minnesota	PENUT Site Co-I	
Bradley	Yoder		MD	University of Utah	Salt Lake City, Utah	PENUT Site Co-I	
Jordan S.	Kase		MD	Maria Fareri Children's Hospital at Westchester	Valhalla, New York	PENUT Site Co-I	
Jennifer	Check		MD, MS	Wake Forest School of Medicine	Winston-Salem, North Carolina	PENUT Site Co-I	
Erin	Osterholm		MD	University of Minnesota Masonic Children's Hospital	Minneapolis, Minnesota	PENUT Site Co-I	
Thomas	George		MD	University of Minnesota Masonic Children's Hospital	Minneapolis, Minnesota	PENUT Site Co-I	
Michael	Georgieff		MD	University of Minnesota Masonic Children's Hospital	Minneapolis, Minnesota	PENUT Site Co-I	
Camilia R.	Martin		MS	Beth Israel Deaconess Medical Center	Boston, Massachusetts	PENUT Site Co-I	

*Indicates required information. Only first name, last name, and suffix will appear in PubMed.

***First Name and Middle Initial(s)**	***Last Name**	***Suffix (eg, Jr, III)**	Academic Degrees	Institution	Location (city, state/province, country)	Role or Contribution, eg, chair, principal investigator	Group (if more than 1 Group listed in the byline) and/or Subgroup (eg, Steering Committee)
Deirdre	O'Reilly		MD, MPH	Beth Israel Deaconess Medical Center	Boston, Massachusetts	PENUT Site Co-I	
Raye-Ann	deRegnier		MD	Prentice Women's Hospital	Chicago, Illinois	PENUT Site Co-I	
Nicolas	Porta		MD	Prentice Women's Hospital	Chicago, Illinois	PENUT Site Co-I	
Catalina	Bazacliu		MD	University of Florida	Gainesville, Florida	PENUT Site Co-I	
Frances	Northington		MD	Johns Hopkins University	Baltimore, Maryland	PENUT Site Co-I	
Raul	Chavez Valdez		MD	Johns Hopkins University	Baltimore, Maryland	PENUT Site Co-I	
Patel	Saurabhkumar		MD, MPH	Children's Hospital of the University of Illinois	Chicago, Illinois	PENUT Site Co-I	
Magaly	Diaz-Barbosa		MD	South Miami Hospital	South Miami, Florida	PENUT Site Co-I	
Arturo E.	Serize		MD	South Miami Hospital	South Miami, Florida	PENUT Site Co-I	
Jorge	Jordan		MD	South Miami Hospital	South Miami, Florida	PENUT Site Co-I	
John	Widness		MD	University of Iowa,	Iowa City, Iowa	Independent Medical Monitor	
Adam	Hartman		MD	National Institute of Neurological Disorders and Stroke	Bethesda, MD	PENUT Executive Committee	
Roberta	Ballard		MD	University of California	San Francisco, CA	PENUT Executive Committee	
Michael	O'Shea		MD	University of North Carolina School of Medicine	Chapel Hill, NC	PENUT Executive Committee	
Christopher	Nefcy		BS	University of Washington	Seattle, WA	Senior Computer Programmer	
Mark A.	Konodi		MS	University of Washington	Seattle, WA	Biostatistician	
Phuong T.	Vu		PhD	University of Washington	Seattle, WA	Biostatistician	
John B.	Feltner		MS	University of Washington	Seattle, WA	Project Manager	
Isabella	Esposito		BS	University of Washington	Seattle, WA	Research Coordinator	
Stephanie	Hauge		MS	University of Washington	Seattle, WA	Research Coordinator	
Samantha	Nikirk		MPH	University of Washington	Seattle, WA	Research Coordinator	
Amy	Silvia		MS	University of Washington	Seattle, WA	Research Coordinator	

Supplemental Online Content: Nonauthor Collaborators

*Indicates required information. Only first name, last name, and suffix will appear in PubMed.

***First Name and Middle Initial(s)**	***Last Name**	***Suffix (eg, Jr, III)**	Academic Degrees	Institution	Location (city, state/province, country)	Role or Contribution, eg, chair, principal investigator	Group (if more than 1 Group listed in the byline) and/or Subgroup (eg, Steering Committee)
Bailey	Clopp		BS	University of Washington	Seattle, WA	Research Coordinator	
Debbie	Ott		RNC-NIC	Advent Health for Children	Orlando, Florida	Research Coordinator	
Ariana	Franco Mora		BS	Advent Health for Children	Orlando, Florida	Research Coordinator	
Pamela	Hedrick		BA, ASN, RI	Advent Health for Children	Orlando, Florida	Research Coordinator	
Vicki	Flynn		BA, ASN, RI	Advent Health for Children	Orlando, Florida	Research Coordinator	
Andrea	Wyatt		RN, BSN	University of Arkansas for Medical Sciences	Little Rock, Arkansas	Research Nurse	
Emilie	Loy		RN, BSN	University of Arkansas for Medical Sciences	Little Rock, Arkansas	Research Nurse	
Natalie	Sikes		BSN, MSN	University of Arkansas for Medical Sciences	Little Rock, Arkansas	Research Nurse	
Melanie	Mason		RN	University of Arkansas for Medical Sciences	Little Rock, Arkansas	Research Nurse	
Jana	McConnell		RN	University of Arkansas for Medical Sciences	Little Rock, Arkansas	Research Nurse	
Tiffany	Brown		BS	University of Arkansas for Medical Sciences	Little Rock, Arkansas	Research Coordinator	
Henry	Harrison		RN	University of Arkansas for Medical Sciences	Little Rock, Arkansas	Research Nurse	
Denise	Pearson		RN	University of Arkansas for Medical Sciences	Little Rock, Arkansas	Research Nurse	
Tammy	Drake		RN	University of Arkansas for Medical Sciences	Little Rock, Arkansas	Research Nurse	
Jocelyn	Wright		RN	University of Arkansas for Medical Sciences	Little Rock, Arkansas	Research Nurse	
Debra	Walden		RN	University of Arkansas for Medical Sciences	Little Rock, Arkansas	Research Nurse	
Annette	Guy		RN	University of Arkansas for Medical Sciences	Little Rock, Arkansas	Research Nurse	
Jennifer	Nason		RN	University of Louisville	Louisville, Kentucky	Research Nurse	
Morgan	Talbot		BSN	University of Louisville	Louisville, Kentucky	Research Nurse	

Supplemental Online Content: Nonauthor Collaborators

*Indicates required information. Only first name, last name, and suffix will appear in PubMed.

*First Name and Middle Initial(s)	*Last Name	*Suffix (eg, Jr, III)	Academic Degrees	Institution	Location (city, state/province, country)	Role or Contribution, eg, chair, principal investigator	Group (if more than 1 Group listed in the byline) and/or Subgroup (eg, Steering Committee)
Kristen	Lee		BS	University of Louisville	Louisville, Kentucky	Research Coordinator	
Sarah	Penny		BA	University of Louisville	Louisville, Kentucky	Regulatory Manager	
Terri	Boles		BA	University of Louisville	Louisville, Kentucky	Regulatory Assistant	
Melanie	Drummond		RN, BSN	Methodist Children's Hospital	San Antonio, Texas	Research Nurse	
Katy	Kohlleppel		RN, BSN	Methodist Children's Hospital	San Antonio, Texas	Research Nurse	
Charmaine	Kathen		BSN, MSN,	Methodist Children's Hospital	San Antonio, Texas	Research Coordinator	
Brian	Kaletka		BA	Children's Minnesota	Minneapolis, Minnesota; St. Paul, Minnesota	Research Coordinator	
Shania	Gonzales		BS	Children's Minnesota	Minneapolis, Minnesota; St. Paul, Minnesota	Research Coordinator	
Cathy	Worwa		BS	Children's Minnesota	Minneapolis, Minnesota; St. Paul, Minnesota	Research Coordinator	
Molly	Fisher		BS	Children's Minnesota	Minneapolis, Minnesota; St. Paul, Minnesota	Research Coordinator	
Tyler	Richter		BS	Children's Minnesota	Minneapolis, Minnesota; St. Paul, Minnesota	Research Coordinator	
Alexander	Ginder		BS	Children's Minnesota	Minneapolis, Minnesota; St. Paul, Minnesota	Research Coordinator	
Brixen	Reich		RN	University of Utah	Salt Lake City, Utah	Research Nurse	
Carrie	Rau		RN	University of Utah	Salt Lake City, Utah	Research Nurse	
Manndi	Loertscher		BS	University of Utah	Salt Lake City, Utah	Research Coordinator	
Laura	Cole		RN	University of Utah	Salt Lake City, Utah	Research Nurse	
Kandace	McGrath		AS	University of Utah	Salt Lake City, Utah	Research Coordinator	
Kimberlee	Weaver Lewis		BSN, MSN	University of Utah	Salt Lake City, Utah	Research Nurse	

Supplemental Online Content: Nonauthor Collaborators

*Indicates required information. Only first name, last name, and suffix will appear in PubMed.

***First Name and Middle Initial(s)**	***Last Name**	***Suffix (eg, Jr, III)**	Academic Degrees	Institution	Location (city, state/province, country)	Role or Contribution, eg, chair, principal investigator	Group (if more than 1 Group listed in the byline) and/or Subgroup (eg, Steering Committee)
Jill	Burnett		BSN, RNC-N	University of Utah	Salt Lake City, Utah	Research Nurse	
Susan	Schaefer		RRT, RN, BS	University of Utah	Salt Lake City, Utah	Research Nurse	
Karie	Bird		RN	University of Utah	Salt Lake City, Utah	Research Nurse	
Clare	Giblin		BSN, RNC	Maria Fareri Children's Hospital	Valhalla, New York	Research Nurse	
Rita	Daly		BA	Maria Fareri Children's Hospital	Valhalla, New York	Grants Manager	
Kristi	Lanier		RN, BSN	Wake Forest School of Medicine	Winston-Salem, North Carolina	Research Nurse	
Kelly	Warden		RN, BSN	Wake Forest School of Medicine	Winston-Salem, North Carolina	Research Nurse	
Jenna	Wassenaar		BS	University of Minnesota Masonic Children's Hospital	Minneapolis, Minnesota	Research Coordinator	
Jensina	Ericksen		RN, BSN	University of Minnesota Masonic Children's Hospital	Minneapolis, Minnesota	Research Nurse	
Bridget	Davern		BS	University of Minnesota Masonic Children's Hospital	Minneapolis, Minnesota	Research Coordinator	
Brittany	Gregorich		BA, MPH	University of Minnesota Masonic Children's Hospital	Minneapolis, Minnesota	Research Coordinator	
Mary Pat	Osborne		BS, MSN	University of Minnesota Masonic Children's Hospital	Minneapolis, Minnesota	Research Nurse	
Neha	Talele		BA, MPH	Beth Israel Deaconess Medical Center	Boston, Massachusetts	Research Coordinator	
Evelyn	Obregon		MD	Beth Israel Deaconess Medical Center	Boston, Massachusetts	Research Fellow	
Tiglath	Ziyeh		BS, MS	Beth Israel Deaconess Medical Center	Boston, Massachusetts	Research Coordinator	
Molly	Clarke		BS	Beth Israel Deaconess Medical Center	Boston, Massachusetts	Research Coordinator	
Rachel E	Wegner		BA, MS, MS	Beth Israel Deaconess Medical Center	Boston, Massachusetts	Research Program Manager	

Supplemental Online Content: Nonauthor Collaborators

*Indicates required information. Only first name, last name, and suffix will appear in PubMed.

***First Name and Middle Initial(s)**	***Last Name**	***Suffix (eg, Jr, III)**	Academic Degrees	Institution	Location (city, state/province, country)	Role or Contribution, eg, chair, principal investigator	Group (if more than 1 Group listed in the byline) and/or Subgroup (eg, Steering Committee)
Palak	Patel		BS	Beth Israel Deaconess Medical Center	Boston, Massachusetts	Research Coordinator	
Molly	Schau		RN, BSN	Prentice Women's Hospital	Chicago, Illinois	Research Nurse	
Annamarie	Russow		BS	Prentice Women's Hospital	Chicago, Illinois	Research Coordinator	
Kelly	Curry		RN, MSN	University of Florida	Gainesville, Florida	Research Nurse	
Lisa	Barnhart		RNC-NIC	University of Florida	Gainesville, Florida	Research Nurse	
Charlamaine	Parkinson		BS, BSN, M	Johns Hopkins University	Baltimore, Maryland	Research Nurse	
Sandra	Beauman		BSN, MSN,	University of New Mexico	Albuquerque, New Mexico	Co-Investigator and Research Nurse	
Mary	Hanson		RN, BSN	University of New Mexico	Albuquerque, New Mexico	Research Nurse	
Elizabeth	Kuan		RN, BSN	University of New Mexico	Albuquerque, New Mexico	Research Nurse	
Conra	Backstrom Lacy		RN	University of New Mexico	Albuquerque, New Mexico	Research Nurse	
Edshelee M.	Galvis		BA	South Miami Hospital	South Miami, Florida	Research Coordinator	
Susana	Bombino		BA	South Miami Hospital	South Miami, Florida	Research Coordinator	
Denise	Martinez		BA, MPH	Johns Hopkins All Children's Hospital	St. Petersburg, Florida	Research Coordinator	
Suzi	Bell		BSN, MSN,	Johns Hopkins All Children's Hospital	St. Petersburg, Florida	Research Nurse	
Corrie	Long		RN, BSN	Johns Hopkins All Children's Hospital	St. Petersburg, Florida	Research Nurse	
